# Biomechanics of Subcutaneous Locked Plating Versus Burke Plate and External Fixator for Comminuted Distal Radius Fractures

**DOI:** 10.7759/cureus.39142

**Published:** 2023-05-17

**Authors:** Dominik Fleifel, Andrew V Pytiak, Xin Jin, Zlatan Cizmic, Rahul Vaidya

**Affiliations:** 1 Department of Orthopaedic Surgery, Wayne State University School of Medicine, Detroit, USA; 2 Department of Orthopaedic Surgery, Detroit Medical Center, Detroit, USA; 3 Department of Pediatric Orthopaedic Surgery, Rocky Mountain Hospital for Children, Denver, USA; 4 Department of Biomedical Engineering, Wayne State University School of Medicine, Detroit, USA; 5 Department of Orthopaedic Surgery, St. John Providence Hospital, Southfield, USA

**Keywords:** burke plate, fracture, radius, subcutaneous, external fixator, internal fixation

## Abstract

Background

External fixators that span the wrist have been the historical norm in treating distal radius fractures. We have modified a dorsal distraction approach by using a subcutaneously applied locked bridge plate through two small incisions superficial to the extensor tendons and outside the extensor compartment. The purpose of this study was to biomechanically evaluate this modified method of fixation for comminuted distal radius fractures in comparison with two established constructs.

Methods

Matched cadaver specimens were used to model an AO Type 23-C3 distal radius fracture. Biochemical testing for stiffness during axial compressive loading was done on three constructs: a conventional Burke distraction plate, the subcutaneous internal fixation plating technique, and an external fixator. All specimens were cyclically loaded for 3000 cycles and then retested.

Results

The modified construct was found to be stiffer than the external fixator (p=0.013). When compared to the Burke plate, the modified construct was significantly less stiff before axial cycling (p=0.025). However, the difference was not maintained after cycling, and the post-axial loading stiffness difference was non-significant (p=0.456).

Conclusion

Our data demonstrate the biomechanical integrity of the subcutaneous plating technique for the fixation of comminuted distal radius fractures. It is stiffer than an external fixator and has the theoretical advantage of avoiding pin-tract infections. In addition, it is subcutaneous and not a cumbersome external construct. Our construct is minimally invasive, and it does not violate the dorsal extensor compartments. This allows for finger movement even while the construct is in place.

## Introduction

Distal radius fractures are extremely common injuries, comprising the plurality of fractures seen in the emergency room in the United States. Due to the debilitating nature of these fractures, a large proportion require surgical management. Comminuted fractures of the distal radius have proven particularly difficult to reduce and stabilize [1-4].

While still used for most severely comminuted intraarticular fractures, external fixators can have inadequate maintenance of reduction as well as complications associated with pin tract infection, superficial radial nerve neuropathy, pin loosening, and stiffness [5-9]. The complications associated with external fixation of highly comminuted and multiple injury radial fractures has left these techniques as amenable treatment options for low-demand patients [10]. 

Internal fixation with plating provides adequate stabilization without the complications of external fixators [11,12]. Burke and Singer first described the dorsal distraction plate which has been used by several authors for addressing severely comminuted fractures that are not amenable to traditional open reduction and internal fixation [11,13]. The distraction plate aims to reduce immobilization, increasing tensile strength and physical demand on ligaments, resulting in more effective stabilization and recovery [3]. This technique not only minimizes complications, but provides more construct stiffness than external fixation [14]. However, the distal radius spanning distraction plate has some associated morbidity, concerning adhesion and rupture of the extensor pollicis longus, nerve entrapment, and hardware failure [15]. In addition to these risks, studies have found a 10° extensor lag [11]. Recently, Lewis et al. have found that conventional plating onto the 3rd metacarpal has a significant increase in adverse effects compared to insertion onto the 2nd metacarpal [16]. We propose an adaptation of this distal radius spanning technique that places the plate subcutaneously, while staying superficial to the extensor compartment and plating to the 2nd metacarpal or 3rd metacarpal, thereby addressing the concerns of tendon damage, muscle disruption, and restricted finger movement. By doing so, reduction and stabilization is provided to a severely comminuted radial fracture without disrupting the muscle tendons; theoretically allowing improved retention of finger range of motion in the postoperative period, ease of insertion and removal, and minimal soft tissue or compartment disruption. While there has been biomechanical and clinical evidence to support the superiority of plating to external fixators, biomechanical testing of a subcutaneous plate has not been published to the authors’ knowledge at the time of this article [17].

Open reduction and internal fixation is the predominant method of fracture fixation of the distal radius [18-20]. Plating has reduced the rate of infections and provided patients with the ability to bear weight on the extremity. However, it comes with the risk of tendon compartment entrapment or tendon lag [11,16]. Recently, Lauder A et al. conducted a retrospective study that analyzed the functional outcomes of dorsal-spanning bridge plating. They found that grip strength was 86% and extension torque was 78% compared to the uninjured contralateral wrist. Dominant and non-dominant wrist injuries were identified with nearly complete recovery of dominant-sided grip (95%) and extension (96%) and non-dominant-sided grip (79%) and extension (65%) strength. There were only two cases of postoperative surgical site pain with no cases of infection, tendonitis, or tendon rupture [21]. However, this is not to say that dorsal-spanning bridge plating lacks complications. Such complications can be separated into minor (wound healing and hardware failure without loss of reduction) and major complications (malunion, nonunion requiring surgery, wound complications/unplanned surgery, deep infection, extensor tendon adhesions/tenolysis, extensor pollicis longus muscle (EPL) rupture requiring an extensor indicis proprius (EIP) transfer) [22]. Several studies have demonstrated clinical complications with the use of the conventional distraction plate as well. Hanel DP et al. reported one extensor carpi radialis longus to rupture with hardware failure in their study of 52 patients 22. In their study, Ruch DS et al. reported that three of 22 patients had extensor lags of 10°. The mean disabilities of the arm, shoulder and hand (DASH) score for these patients was 11.5 points at the final follow-up [11]. Richard MJ et al. reviewed distraction plating of comminuted distal radius fractures in 33 elderly patients and reported complications, including digital stiffness, required tenolysis at the time of plate removal, superficial radial neuritis, and chronic regional pain syndrome [23]. Another study on the dorsal-bridge plating technique identified complications, including tendon rupture, tenosynovitis, reoperation, and 25% collapse [24]. Additional drawbacks of subcutaneously placed dorsal plating include the prominence of the hardware, especially in thin patients, and associated irritation from the plate.

The modified Burke plate offers the potential to provide quicker recovery of finger range of motion, decreased risk of infection, minimal soft tissue damage upon insertion and removal, and a less visible scar. The purpose of this study was to evaluate the biomechanical aspects of the modified internal fixation plate for comminuted distal radius fractures in comparison with two established constructs that are commonly used in similar radial fractures. We hypothesize that our novel subcutaneous locked bridge plate will be stiffer than the external fixator device and less stiff than the Burke plate.

This article was previously posted to the Research Square preprint server on October 21, 2020.

## Materials and methods

**Figure 1:**
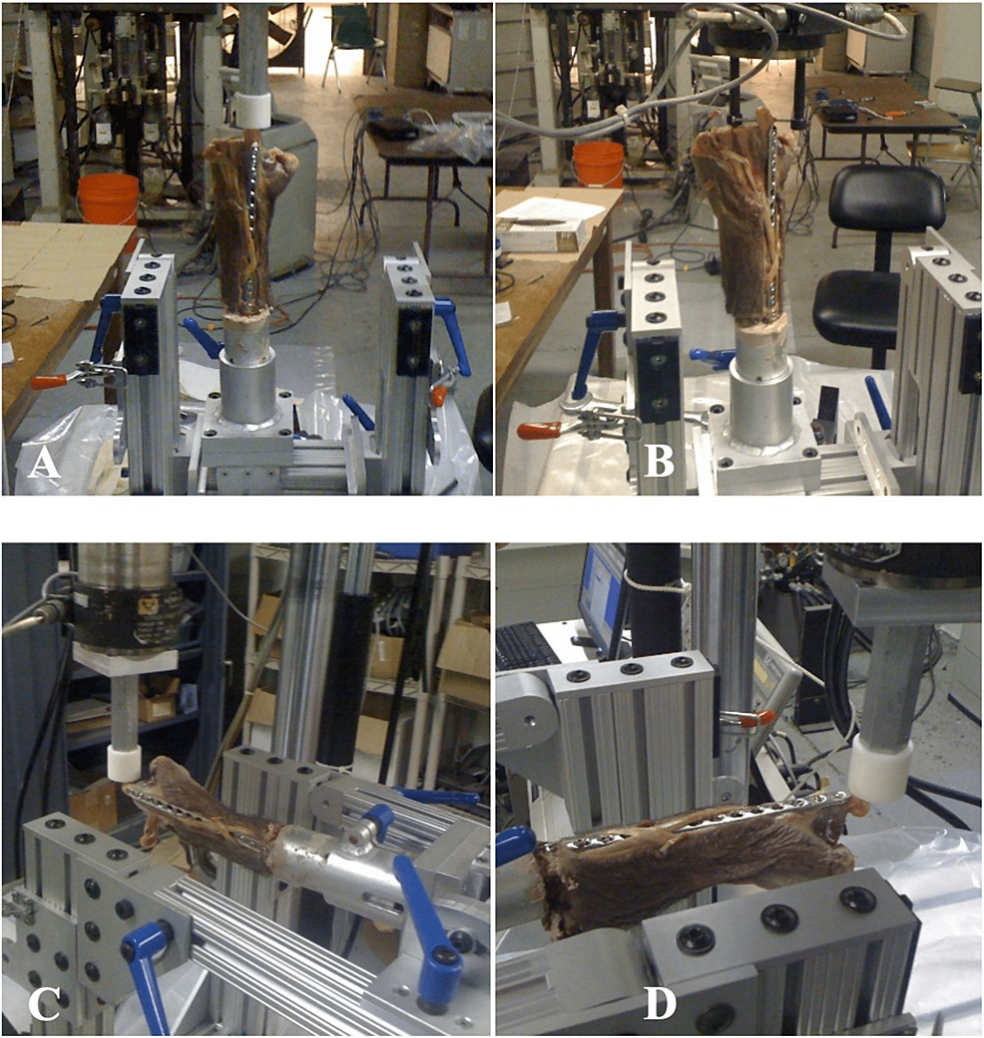
Testing positions and point of loading of the specimens within the materials testing machine for A) axial loading, B) torsional loading, C) radial-to-ulnar loading, and D) dorsal-to-volar loading.

Three sets of age-match paired fresh frozen cadavers were obtained for testing. Three constructs were tested: a 3.5 mm 14-hole plate (Synthes, West Chester, PA) placed using the subcutaneous plating technique, a 3.5 mm 14-hole plate (Synthes, West Chester, PA) using the Burke internal distraction technique, and a standard external fixation construct which spanned the distal radius. Each construct was tested on three matched pairs of radii. Each technique was first assembled on the respective radius, followed by a 1 cm transverse osteotomy of the distal radius to create a defect in the distal radius to simulate a severely comminuted fracture. This approach aimed to eliminate any bias between fixation and create an ideal comparison available for biomechanical testing. Biomechanical comparisons were evaluated for axial compression loading, cantilever bending in volar-to-dorsal, dorsal-to-volar, ulnar-to-radial, radial-to-ulnar directions, and torsion loading. A materials testing system (Instron 8500, Norwood, MA) was used to test each specimen under load control. Specimens were all cyclically loaded at 50N. Torsional loading was performed at 0.5 N/m. Three thousand cycles were applied in each direction to every construct. Specimens were tested in the following order: axial compression, volar-to-dorsal cantilever bending, dorsal-to-volar cantilever bending, radial-to-ulnar cantilever bending, ulnar-to-radial cantilever bending, and torsional loading. Displacement was measured in millimeters with respect to the load for each specimen. The specimens were mounted on a custom jig after being potted in polymethylmethacrylate (Figure 1). Following potting and setup in the custom jig, axial loading was applied on the distal aspect of the second metacarpal head. For the point of bending, torque was directly applied through a pin used for stabilization of the second metacarpal into the custom jig.

Surgical techniques

**Figure 2:**
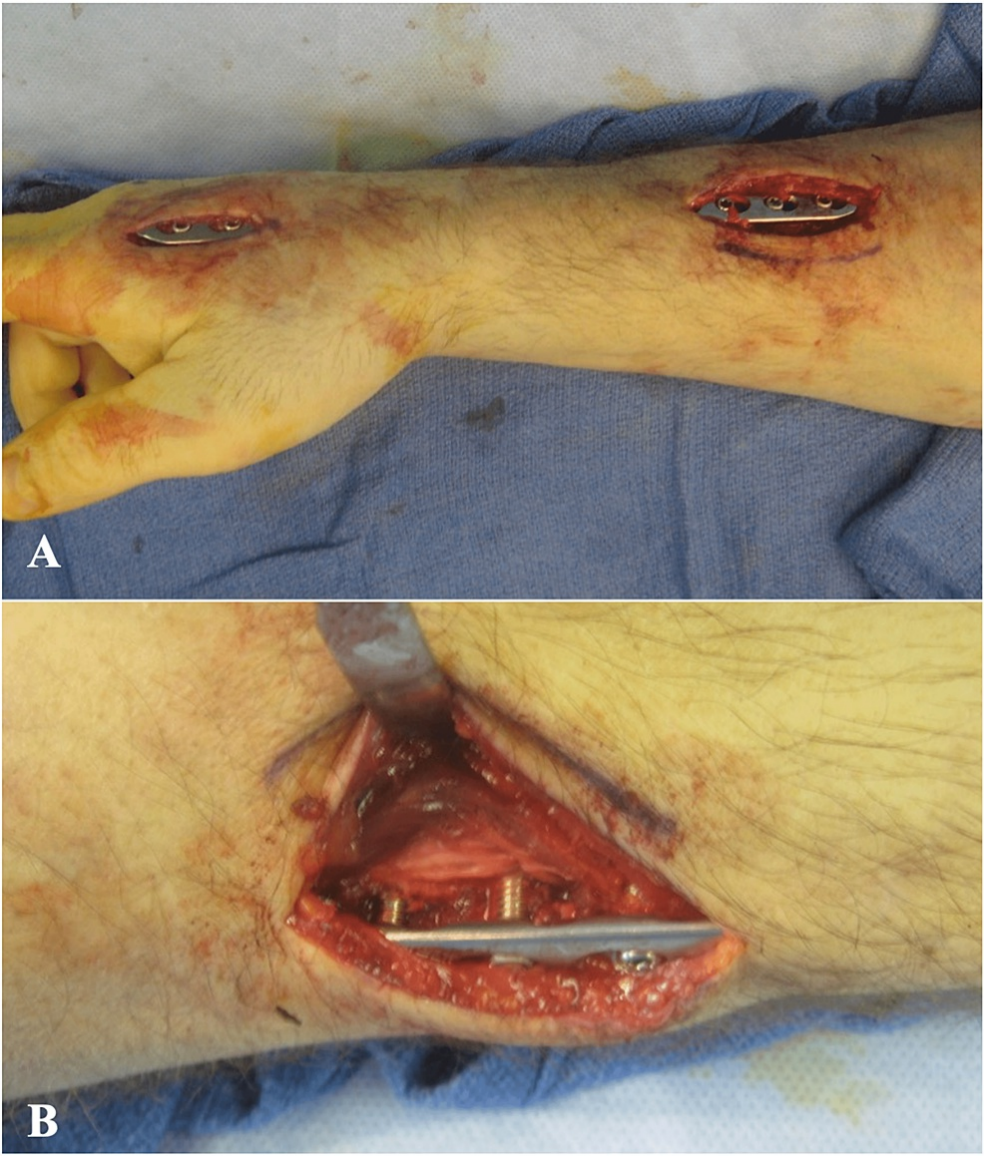
Photograph of fixation demonstrating A) incision placements for subcutaneous in-fix plate fixation along the dorsal aspect of the forearm and B) a close-up of the proximal incision demonstrating its subcutaneous placement and suspension superficial to the fascia and extensor compartments.

The internal distraction plate was placed as previously described by Burke EF and Singer RM [13]. Dissection was performed over the third compartment, and the EPL was mobilized to expose the bone fragment in which the defect would be created. Dissection was carried distally over the 2nd metacarpal to provide an adequate comparison with the subcutaneous infix plate. The extensor tendon was identified and protected while exposing the 2nd metacarpal. The 3.5 mm, 14-hole Synthes plate was then aligned from the dorsal surface of the distal radius, just ulnar to the extensor carpi radialis longus and brevis, to the 2nd metacarpal, deep to the extensor tendons as described by Burke EF and Singer RM [11,13]. The plate was then fixed proximally and distally with three non-locking cortical screws on either side of the osteotomy site. The second construct that was tested was the subcutaneous infix distal radius spanning technique. The infix technique also involved using a 14-hole, 3.5 mm locking plate. Two small incisions on the dorsal aspect of the arm were made, the first between the 2nd and 3rd metacarpal and the second overlying the distal third of the radius (Figure 2). Blunt dissection was performed, connecting the two incisions while staying superficial to the extensor tendons and outside the third and fourth extensor compartments. The plate was then slid subcutaneously from the proximal incision to the distal incision. The plate was fixed subcutaneously on the dorsal aspect of the forearm spanning from the radius to the second metacarpal with three locking screws on either side of the osteotomy site. In order to ensure similar testing conditions, all subcutaneous plates were placed 15 mm off of the bone.

A small external fixator (Synthes, West Chester, PA) was the third construct that was tested. Percutaneous pins were inserted through the second and third metacarpal bones and the radius in a transverse direction. A low-torque power drill was used to insert the Steinmann pins. The distal pins were placed transverse and perpendicular to each other at the bases of the second and third metacarpals. This was followed by drilling parallel pins to the distal radius approximately 3-5 centimeters proximal to the planned osteotomy site. Once each construct was secured, a transverse osteotomy was performed on the distal radius to create a 1 cm defect to simulate a severely comminuted distal radius fracture. All specimens, with fixed constructs already attached, were potted and inserted into a custom jig prior to performing the osteotomy.

Statistical analysis

Mann-Whitney U comparisons were performed in order to compare pre-axial to post-axial loading conditions. Nonparametric analysis was chosen, given the small sample size. Alpha value was set at 0.05; an assumption was made of superiority to external fixation and non-inferiority compared to the Burke plate. Nonparametric and descriptive analyses were performed with SAS 9.3 (SAS Institute Inc, Cary, NC).

## Results

The Burke plate was found to have the following average post-cyclic loading stiffnesses: 299.93 N/m during axial loading, 4.36 N/m during radial-to-ulnar bending, 4.25 N/m during ulnar-to-radial, 1.99 N/m dorsal-to-volar bending, 1.39 N/m during volar-to-dorsal bending, and 0.03 Nm/m during torsional loading. No failure was noted during cyclic loading. The custom subcutaneous plate was found to have the following average post-cyclic loading stiffnesses: 217.83 N/m during axial loading, 3.25 N/m during radial-to-ulnar bending, 3.25 N/m during ulnar-to-radial bending, 1.21 N/m dorsal-to-volar bending, 1.02 N/m during volar-to-dorsal bending, and 0.03 Nm/m during torsional loading. No failure was noted during cyclic loading. The external fixation construct was found to have the following average post-cyclic loading stiffnesses: 49.33 N/m during axial loading, 1.63 N/m during radial-to-ulnar bending, 1.83 N/m during ulnar-to-radial bending, 1.91 N/m dorsal-to-volar bending, 1.77 N/m during volar-to-dorsal bending, and 0.06 Nm/m during torsional loading. No failure was noted during cyclic loading. In terms of axial stiffness, the modified subcutaneous construct was found to be stiffer than the external fixator after load cycling (p=0.013). When comparing the Burke plate to the modified subcutaneous construct, there was no significant difference in stiffness at the end of cyclic loading (p=0.456) (Table 1).

**Table 1:** Measured stiffness of the three constructs: external fixator, Burke plate, and subcutaneous plate.

	External Fixator (Ex-fix)	Burke Plate	Subcutaneous Plate	P-value (Ex-fix versus subcutaneous plate)	P-value (Burke plate versus subcutaneous plate)
Post-cycling axial loading	49.33 N/m	299.93 N/m	217.83 N/m	0.013	0.456
Radial-to-ulnar loading	1.63 N/m	4.36 N/m	3.25 N/m	N/A	N/A
Ulnar-to-radial loading	1.83 N/m	4.25 N/m	3.25 N/m	N/A	N/A
Dorsal-to-volar loading	1.91 N/m	1.99 N/m	1.21 N/m	N/A	N/A
Volar-to dorsal loading	1.77 N/m	1.39 N/m	1.02 N/m	N/A	N/A
Torsional Loading	0.06 Nm/m	0.03 Nm/m	0.03 Nm/m	N/A	N/A

## Discussion

There has been controversy in the specific technical approaches between volar and dorsal plating. Some authors prefer a volar plate to avoid the risk of extensor tendon damage, while others believe that a volar approach provides better biomechanical outcomes [25-35]. Rein S et al. described two of 14 patients with dorsal plating who experienced extensor tendon irritation, two patients with fragment displacement, and three cases of nerve irritation. This is compared to 12 of 14 patients with no complications with the volar approach. However, they also point out that novel dorsal plates and modified techniques could overcome these issues [34]. Similarly, Henry MH et al. described the advantages of volar plating as having a socially less visible scar and the ability to support immediate functional loading of the hand, wrist, and forearm in rehabilitation and daily activities. It was also noted that with increasing fracture complexity, restoration of volar tilt becomes increasingly difficult without the use of a device to achieve the lifting maneuver [29]. Radiologic, clinical, and biomechanical studies have been performed, advocating the superiority of dorsal plating over volar plating. Letsch R et al. provided radiologic evidence that palmar plates (1.7 points) had a significantly lower result than patients with a dorsal plate (1.0 points, p< 0.05). In such studies, locked or non-locked dorsal constructs were more than two times stiffer than volar constructs. With regards to functional outcome, there was a strong tendency for better outcomes with dorsal plating (3.0 points) compared with palmar plating (4.1 points); however, these findings were not significant [36]. A biomechanical study by Trease C et al. proved that while not statistically significant, the failure strength of dorsal constructs was 53% higher than that of volar constructs [37]. While volar versus dorsal plating remains an ongoing issue, the novel subcutaneous plate provides stiffness as good as the conventional dorsal-spanning bridge plate, therefore handling stiffness similar to volar plates. The modified subcutaneous construct aims to overcome common complications of severely comminuted distal radius fractures via multiple modifications to current techniques. Lewis S et al. first provided evidence that plating to the second metacarpal did not result in tendon entrapment, but plating to the third metacarpal resulted in six tendon entrapments in six samples [16]. With respect to Lewis' data for the insertion of distal plating sites, the modified construct was placed on the 2nd metacarpal to minimize tendon entrapment. In addition, by staying superficial to the extensor compartment, the complications, as mentioned earlier, should theoretically be marginal due to minimal soft tissue disruption and violation of the third and fourth extensor compartments, as well as bypassing possible nerve injury. The data from the current study suggest that the subcutaneous internal fixator plate provides adequate structural support similar to the traditional Burke approach after cycling and is both stiffer and stronger than an external fixator.

In the current study, we tested the axial and torsional durability of external fixation, the Burke plate, and subcutaneous plating. The results demonstrate the biomechanical integrity of our modified construct for the fixation of comminuted distal radius fractures. It is stiffer than an external fixator and has the theoretical advantage of avoiding pin-tract infections. In addition, it is subcutaneous and less cumbersome than the external construct. Our construct is minimally invasive, easier to remove, and does not violate the dorsal extensor compartments, allowing movement of the fingers while the construct is in place. The results of this study, in addition to the theoretical circumvention of well-known complications, support dorsal subcutaneous plating as an alternative for highly comminuted distal radius fractures. While the current study portrays biomechanical loading as good as the conventional plate and has better stiffness than the external fixator, the current study also has limitations. A primary limitation of this study is the relatively low number of specimens used. However, nonparametric statistical analyses are less sensitive to sample size issues, and statistical significance was still achieved despite the limited number of specimens used. This study was performed in cadaveric specimens; therefore, the effects of osteogenesis on the tested constructs could not be evaluated, and cyclic data should be interpreted cautiously. Finally, while age-matched specimens allowed for the comparison of two biologically similar samples, the clinical significance of these results cannot be determined without further study. We accept the alternative hypothesis that the modified subcutaneous construct is superior to external fixation in terms of stiffness in both pre-and post-axial load cycling. We found that the Burke plate was superior in stiffness before axial cycling. However, the difference does not reach significance after axial cycling, suggesting that the modified plate should be as resilient as the Burke plate in actual use. We recognize that the small sample size may have affected the power to detect a post-cycling difference. However, based on marked superiority to external fixation and reduced invasiveness for installation, we have identified an essential alternative technique for the fixation of severely comminuted distal radius fractures. Future studies will focus on the clinical results of the subcutaneous infix plating technique.

## Conclusions

The modified subcutaneous internal fixator device provides superior stiffness results compared to the external fixator in pre- and post-axial cycling. The construct is less invasive than the external fixator while having the potential to limit commonly associated infections. In this data series, the subcutaneous internal fixator device exhibited significantly inferior stiffness pre-axial cycling and nonsignificant inferior stiffness post-axial cycling as compared to the Burke plate.
